# Questions on travel and sexual behaviours negatively impact ethnic minority donor recruitment: Effect of negative word‐of‐mouth and avoidance

**DOI:** 10.1111/vox.13748

**Published:** 2024-11-06

**Authors:** Eamonn Ferguson, Richard Mills, Erin Dawe‐Lane, Zaynah Khan, Claire Reynolds, Katy Davison, Dawn Edge, Robert Smith, Niall O'Hagan, Roshan Desai, Mark Croucher, Nadine Eaton, Susan R. Brailsford

**Affiliations:** ^1^ School of Psychology University of Nottingham Nottingham United Kingdom; ^2^ National Institute for Health and Care Research Blood and Transplant Research Unit in Donor Health and Behaviour University of Cambridge Cambridge United Kingdom; ^3^ Clinical, Educational and Health Psychology, Psychology and Language Sciences University College London London United Kingdom; ^4^ Research Support Officer, Institute of Mental Health, Nottinghamshire Healthcare NHS Foundation Trust United Kingdom; ^5^ NHS Blood and Transplant/UK Health Security Agency Epidemiology Unit NHSBT London United Kingdom; ^6^ NHS Blood and Transplant/UK Health Security Agency Epidemiology Unit UKHSA London United Kingdom; ^7^ Equality, Diversity & Inclusion Research Unit (EDI‐RU), Greater Manchester Mental Health (GMMH) NHS Trust. Biomedical Research Centre Manchester Academic Health Science Centre Manchester United Kingdom; ^8^ NHS Blood and Transplant, Donor Experience Services London United Kingdom; ^9^ NHS Blood and Transplant, Marketing, Transfusions Bristol United Kingdom

**Keywords:** blood donation, donor behaviour, ethnicity, HIV, sexual behaviour, travel

## Abstract

**Background and Objectives:**

Donor selection questions differentially impacting ethnic minorities can discourage donation directly or via negative word‐of‐mouth. We explore the differential impact of two blood safety questions relating to (i) sexual contacts linked to areas where human immunodeficiency virus (HIV) rates are high and (ii) travelling to areas where malaria is endemic. Epidemiological data are used to assess infection risk and the need for these questions.

**Materials and Methods:**

We report two studies. Study 1 is a behavioural study on negative word‐of‐mouth and avoiding donation among ethnic minorities (*n* = 981 people from National Health Service Blood and Transplant (NHSBT) and the general population: 761 were current donors). Study 2 is an epidemiology study (utilizing NHSBT/UK Health Security Agency (UKHSA) surveillance data on HIV‐positive donations across the UK blood services between1996 and 2019) to assess whether the sexual risk question contributes to reducing HIV risk and whether travel deferral was more prevalent among ethnic minorities (2015–2019). Studies 1 and 2 provide complementary evidence on the behavioural impact to support policy implications.

**Results:**

A high proportion of people from ethnic minorities were discouraged from donating and expressed negative word‐of‐mouth. This was mediated by perceived racial discrimination within the UK National Health Service. The number of donors with HIV who the sexual contact question could have deferred was low, with between 8% and 9.3% of people from ethnic minorities deferred on travel compared with 1.7% of White people.

**Conclusion:**

Blood services need to consider ways to minimize negative word‐of‐mouth, remove questions that are no longer justified on evidence and provide justification for those that remain.

**Highlights**

- Donor selection questions on travel and sexual contact linked to human immunodeficiency virus (HIV)‐endemic areas negatively impact ethnic minorities in terms of increased negative word‐of‐mouth and reduced willingness to donate.
- A total of 34% of Black non‐donors decided not to donate because of the sexual contact question, with 17% of Black non‐donors telling others not to. The travel question resulted in 17% of Black non‐donors deciding not to donate and 11.3% telling others not to. These effects were mediated through increased perceived racial discrimination within the National Health Service.
- Surveillance data show that the number of donors with HIV attributed to sex with a higher risk partner from an endemic area is low. Travel questions disproportionately impact ethnic minorities, with 8%–9.3% of people advised not to attend to donate compared with 1.7% of White donors.

## INTRODUCTION

While greater diversity within donor panels is clinically beneficial, disproportionately fewer people from ethnic minorities donate within countries in the global north [1]. One contributory factor we explore is the negative impact of deferral arising from blood donor selection questions differentially impacting ethnic minorities [2]. We explore this negative impact in terms of reduced propensity to donate (avoidance) [2, 3] and negative word‐of‐mouth (_*N*_WoM) [4, 5].

### Consequence of deferral: Personal avoidance and _*N*_WoM

Deferral (i.e., being temporarily or permanently not allowed to donate blood) reduces the likelihood of a person donating again [3] and may have a wider social impact through _*N*_WoM in terms of telling others not to donate [4, 5, 6]. Information through _*N*_WoM is a major concern because it (i) is more believable than positive WoM (_*P*_WoM) (e.g., information that would encourage and support blood donation) [4, 5, 7], (ii) spreads widely and quickly [8] and (iii) is hard to counteract [9, 10]. This is especially true if deferral is perceived as discriminatory and unjust [10]. While the negative impact of _*N*_WoM on productivity is well documented in the business community [4, 5], less is known about _*N*_WoM in the voluntary sector [11] and blood donation in particular [6, 12, 13, 14, 15, 16, 17].

For blood donation, _*P*_WoM encourages (i) recruitment ([6, 13, 14] but see [12]), (ii) positive donor attitudes [15] and (iii) subsequent _*P*_WoM [16]. While _*N*_WoM based on deferral has been reported [17], the impact of _*N*_WoM on blood donation has not been explored. This article addresses this gap in the literature. We explored _*N*_WoM arising from blood donor selection questions that differentially impact ethnic minority communities in the United Kingdom (UK) in 2019, predicting that _*N*_WoM will be higher among ethnic minorities.

In terms of better understanding the mechanisms driving _*N*_WoM for ethnic minorities, we propose that perceived racial discrimination within the UK National Health Service (NHS) and social isolation mediate the link between ethnicity and _*N*_WoM. People from ethnic minorities report greater perceived racial discrimination within health services in the United Kingdom and worldwide [18, 19], and greater social isolation is linked to feelings of marginalization [20, 21]. Thus, enhanced racial discrimination and isolation should foster greater _*N*_WoM by confirming these opinions and experiences [20].

### Awareness: Safety, family and need

We examine three aspects of awareness that should reduce the negative impact of questions: safety, family and need. Awareness that these questions are asked to ensure blood *safety* should mitigate negative impacts by providing a potential justification for their inclusion [22]. Similarly, knowing *family* members who donate should also reduce negative impacts by helping to normalize donation [23, 24]. Finally, awareness of the need for well‐matched blood should also act as a mitigator by reinforcing the importance of the need for a diverse donor pool [19].

### Sex and travel questions in the United Kingdom

We explore the effect of _*N*_WoM and avoidance in two donor selection questions in the UK, which are more likely to impact people from ethnic minorities, using behavioural (Study 1) and epidemiological (Study 2) data collected from UK donors. The first question asked if, in the last 3 months, the potential donor has had sex with anyone who may ever have had sex in parts of the world where human immunodeficiency virus (HIV)/acquired immunodeficiency syndrome (AIDS) is common, including most countries in Africa: Termed the higher risk partner from sub‐Saharan Africa question (HRP‐SSA). If potential donors answered ‘Yes’ to the HRP‐SSA question, they were asked not to donate, although, in England, the deferral could be removed if they had a regular partner willing to give a sample for testing. The second question asked if the potential donor had travelled recently. If they have returned from a tropical area affected by chikungunya, dengue, yellow fever or zika, this resulted in a 1‐month deferral, and returning from the malarious area (e.g., parts of Africa) in a 4‐month deferral followed by additional testing. While these processes, in place for many years, are intended to enhance blood safety, behaviourally, they differentially affect people from ethnic minorities who are more likely to have travelled to Africa, Asia and South America. Indeed, previous findings show that White donors had the lowest proportions of deferral at 2%, with 15% of donors of Indian ethnicity, 10% of Pakistani ethnicity donors and 8% of Black African donors advised not to attend [25].

### Behavioural and epidemiological evidence for policy impacts

Any behavioural impacts, as evidenced by avoidance and _*N*_WoM, would suggest that these questions should be removed or changed. However, the behavioural evidence only tells half the story, and to support policy change, it is also necessary to show no direct impact on donor safety. Therefore, triangulation with epidemiological data is essential. Thus, we explore the effects of the HRP‐SSA question on the incidence of HIV in donors up to 2019 and discuss how many people from ethnic minorities are deferred on the travel question.

### Historical context

The questions examined in this article were in place in 2019 on the donor health check (DHC) at the time. At the time the screening questionnaire in England, Northern Ireland and Scotland were pencil and paper, and in Wales, electronic, but they ask the same questions with slight differences in wording. The work reported in this article contributed to the subsequent removal (HRP‐SSA) or led to an update of pre‐donation information concerning the importance of the travel questions as part of the For the Assessment of Individualised Risk (FAIR) 2 initiative with NHS Blood and Transplant (NHSBT) (https://www.blood.co.uk/news‐and‐campaigns/news‐and‐statements/fair‐steering‐group; NHSBT is a special health authority that is part of the NHS. It is responsible for blood donation services in England) and, as a consequence, has enhanced inclusivity and equity. We report these data to show how triangulation across behaviour and epidemiology data provides robust evidence for policy change. Also, the behavioural data we report here (Study 1) extends previous reports by exploring the mediating role of perceived racial discrimination and social isolation and the social network of donors.

### Aims and hypotheses

We tested the following behavioural hypotheses (Study 1). The HRP‐SSA and travel questions will result in greater reported avoidance and _*N*_WoM in ethnic minorities (H1). People from ethnic minorities will perceive greater racial discrimination within the NHS and greater social isolation (H2). The link between ethnic minorities with avoidance and _*N*_WoM will be mediated by perceived racial discrimination within the NHS (H3a) and social isolation (H3b). Knowing family members who have donated blood (H4a), being aware that the questions are asked to enhance safety (H4b) and being aware of the need for well‐matched blood (H4c) will ameliorate any adverse effects of the HRP‐SSA and travel questions. Using epidemiological and donor management data (Study 2), we (i) examined the number of UK donors with HIV who later reported a potential HRP‐SSA partner and (ii) tested the hypothesis that people from ethnic minorities were more likely to be deferred by the travel question (H5).

## STUDY 1: BEHAVIOURAL EFFECTS ON _*N*_WOM AND AVOIDANCE

### Methods

#### Design and sampling procedure

Six thousand (3500 from ethnic minorities and 2500 from White backgrounds) current donors who had donated within the last 2 years were *randomly* selected from the NHSBT database (ethnicity data were 99% complete in 2019). Non‐donors were recruited through a market research company (Code 3: www.code3research.co.uk: 8600 were randomly selected with 4300 from ethnic minorities and 4300 White people). Initial surveys and reminders were sent out between 14 June 2019 and the 2 August 2019 (see [11] and Supplementary File S1).

##### Coding ethnicity

Self‐described ethnicity was coded using the UK Office of National Statistics (ONS) criteria (Supplementary File S2).

### Materials

Supplementary File S3 provides the survey description, and Supplementary File S4 contains details of the measures.

#### Racial discrimination within the NHS

This was assessed with three items (e.g., ‘Racial discrimination in a doctor's surgery is common’: Supplementary File S4 for all items) (from [26]), summed to give a single scale with higher scores equating to greater discrimination (*α* = 0.84, *M* = 6.75, SD = 2.58, mode = 6, range = 3–15).

#### Social inclusion

We assess this with two items (e.g., ‘Overall, how strongly do you feel about the extent to which you are included in broader society in the UK’: Supplementary File S4 for all items) [27, 28], totalled with higher scores equating to greater social inclusion (*α* = 0.73, *M* = 6.87, SD = 1.82, mode = 8, range = 2–10).

Both the perceived racial discrimination and social isolation questions were scored on a five‐point Likert‐type scale (1 = Strongly Disagree, 2 = Disagree, 3 = Neither Disagree nor Agree, 4 = Agree and 5 = Strongly Agree).

#### Family/community connections with blood donation

We asked, ‘Do you know any people from the following groups who have donated blood?’ (i) your family, (ii) your friends, (iii) your work colleagues and (iv) your neighbourhoods (Yes = 1, No/Don't Know = 0).

#### Awareness of need for ethnic minority blood

We asked, ‘Were you aware that blood from ethnic minority groups is needed to treat diseases like Sickle Cell and Thalassemia? (Yes = 0, No = 1).’

#### Evaluation tasks for the 2019 HRP‐SSA and travel questions

All participants were presented with the following stem:

Before donating blood **everyone** must read an information booklet and complete a form which asks questions about lifestyle, health, and travel. In one question, those presenting to donate blood are asked …

Participants were then presented with the following specific wording for the following:

1. the HRP‐SSA question,

… if in the last three months, they have ‘*had sex with anyone who may ever have had sex in parts of the world where AIDS/HIV is very common (this includes most countries in Africa)?*’ If they answer Yes, they are asked not to donate unless their partner is able to give a sample for testing.

2 the Travel question,

*…* if they have travelled outside the UK in the last 12 months or since their last donation. Specifically, if donors have returned from an area where there is malaria, including many parts of Africa, Asia, and South America in the last 4 months they are asked not to donate.

After reading the HRP‐SSA questions, participants answered questions on awareness and the two primary outcomes of avoidance and _*N*_WoM. This was repeated for the Travel question.

#### Awareness of safety

Participants were asked: ‘This question needs to be asked to keep blood safe for patients’ (*Safety*) and ‘The reason for asking this needs to be explained to the donor’ (*Need*).

#### Primary outcome measures

Participants were also asked: (i) ‘This question would put me off wanting to donate blood’ (*Avoidance*) and (ii) ‘question makes me want to tell others not to donate’ (_*N*_WoM).

Awareness and outcome measures were assessed on a five‐point Likert‐type scale (1 = Strongly Disagree, 2 = Disagree, 3 = Neither Disagree nor Agree, 4 = Agree and 5 = Strongly Agree).

### Analytical strategy

Perceived racial discrimination and social inclusion were normalized using the formulae in Supplementary File S5. As the outcomes are correlated, seemingly unrelated regression (SUR) models accounted for this overlap in the residual error. Models were specified in SPSS‐28 and Stata‐18, with all *p*‐values two‐tailed. Power calculations showed that the sample size provides 80% power (Supplementary File S5).

### Results

The final sample consisted of 981 participants, of which 182 were Asian, 141 Black, 158 mixed ethnicity, 24 other, 456 White and 20 missing. In total, 761 were current donors, 633 were female, 339 were male and 9 were missing, and the mean age was 44.65 years (SD = 14.57) (Supplementary File S2 for full details). There were 719 responses from NHSBT (12% response rate), 254 from code 3 (3% response rate) and 8 from the community sample. Donors were less likely to be Black and women (Table S2 for details).

Hypothesis 1 HRP‐SSA and travel questions result in greater avoidance and _*N*_WoM for ethnic minorities.

**FIGURE 1:**
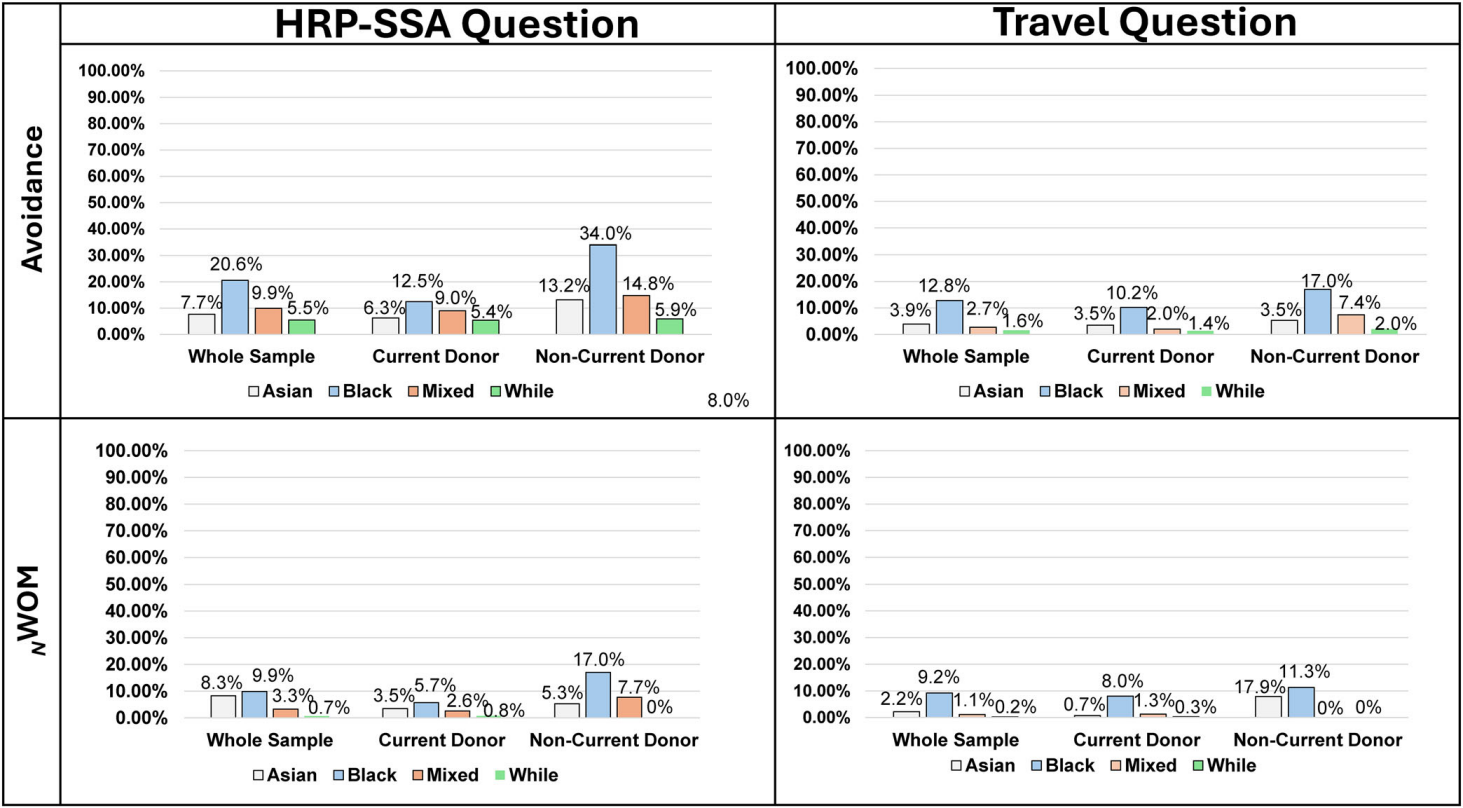
Percentages endorsing strongly agree/agree by ethnicity and donor status. HRP‐SSA, higher risk partner from sub‐Saharan Africa question _*N*_WOM, negative word‐of‐mouth.

To explore H1, we grouped responses for the HRP‐SSA and Travel questions into three combined categories: (i) ‘strongly disagree/disagree’, (ii) ‘neither’ and (iii) ‘strongly agree/agree’. Figure 1 shows the percentage of responses in the ‘strongly agree/agree’ category by ethnicity for the whole sample, current donors and non‐current‐donors (Supplementary File S6 for percentages for all categories by ethnicity and donor experience). For the HRP‐SSA and Travel questions, Black people were significantly more likely to ‘strongly agree/agree’ for ‘avoidance’, reaching 34% of Black non‐donors. Regarding _*N*_WoM, Black people were significantly more likely to endorse ‘strongly agree/agree’ for _*N*_WoM, with this being 17.4% for Black non‐donors. These findings support H1.

Table 1 shows that the predictions from H1 are robust in controlling for demographic factors, safety, need, awareness, discrimination, social inclusion, and family/community connections to blood donation. That is, after reading the HRP‐SSA question, Black people were more likely to say they would avoid donation compared to White people. Similarly, after reading the travel question (Table 1), Black people were more likely to say they would avoid donating compared to White people.

The results in Table 1 also show that following the HRP‐SSA question, avoidance was also higher if people (i) felt that the inclusion of the HRP‐SSA question needed explaining, (ii) were less aware of the need for blood from ethnic minorities, and (iii) knew a family member who has donated blood. Avoidance was lower following the HRP‐SSA question if people (i) believed the question was included to ensure safety and (ii) were current donors. Concerning _*N*_WOM, people from Asian, Black, and Mixed ethnicities are more likely to say that they will tell others not to donate compared to White people. _*N*_WOM was higher if people (i) were older, (ii) felt more socially isolated, and (iii) knew a family member who had donated blood. _*N*_WOM was lower for current donors.

Table 2 also shows that following the travel question, avoidance was higher if people (i) felt that the inclusion of the Travel question needed explaining and (ii) perceived greater racial discrimination within the NHS. Avoidance was lower if people (i) believed the question was included to ensure blood safety and (ii) were current donors. For _*N*_WOM, Asian and Black people were more likely to say that they would tell others not to donate compared to White people. _*N*_WOM was also higher if people (i) were older and (ii) knew a family member had donated blood. _*N*_WOM was lower for current blood donors, and if people felt the question was needed to ensure blood safety.

Hypothesis 2 Greater discrimination within the NHS and social isolation will be observed in ethnic minorities.

**TABLE 1:** Seemingly unrelated regression models for avoidance and negative word‐of‐mouth for sexual behaviour.

	Coefficient	Robust	*p* value	95% CI	
		SE		Lower	Upper
Outcome: avoidance					
Gender (male)	0.0727778	0.0644469	0.259	−0.0535358	0.1990913
Age	0.0026539	0.0026017	0.308	−0.0024454	0.0077532
Need to explain why the question is included	**0.0887023**	**0.0242322**	**0.000**	**0.041208**	**0.1361966**
The question is to ensure safe blood	**−0.5562526**	**0.0481966**	**0.000**	**−0.6507163**	**−0.461789**
Ethnicity (comparison is White)					
Mixed	0.1410763	0.0934134	0.131	−0.0420106	0.3241632
Asian	0.1166425	0.0847232	0.169	−0.049412	0.282697
Black	**0.4077688**	**0.1092357**	**0.000**	0.1936707	0.6218669
Not aware that ethnic blood is needed	**0.1597154**	**0.0779128**	**0.040**	0.007009	0.3124217
Current donor (yes)	**−0.3362416**	**0.0813097**	**0.000**	**−0.4956056**	**−0.1768777**
Social inclusion	−0.244049	0.1418597	0.085	−0.5220889	0.0339909
Racial discrimination within NHS	0.1983081	0.1698497	0.243	−0.1345912	0.5312074
Family member has donated blood (yes)	**0.176632**	**0.0627878**	**0.005**	**0.0535701**	**0.2996939**
Friends has donated blood (yes)	0.0773226	0.0739411	0.296	−0.0675993	0.2222444
Work colleague has donate blood (yes)	−0.0763502	0.0683174	0.264	−0.2102499	0.0575494
Neighbour has donated blood (yes)	−0.0634793	0.084325	0.452	−0.2287533	0.1017947
Constant	**3.84191**	**0.3300852**	**0.000**	**3.194955**	**4.488865**
*R* ^2^	0.29				
Outcome: negative word‐of‐mouth					
Gender (male)	0.0666061	0.0520117	0.200	−0.0353349	0.1685471
Age	**0.0055602**	**0.0021063**	**0.008**	**0.0014318**	**0.0096885**
Need to explain why the question is included	0.0203251	0.0179531	0.258	−0.0148624	0.0555127
The question is to ensure safe blood	**−0.3599031**	**0.0429607**	**0.000**	**−0.4441046**	**−0.2757016**
Ethnicity (Comparison is White)					
Mixed	**0.2015463**	**0.0753653**	**0.007**	0.053833	0.3492597
Asian	**0.2118931**	**0.0676564**	**0.002**	0.079289	0.3444972
Black	**0.4621369**	**0.0899145**	**0.000**	0.2859077	0.6383662
Not aware that ethnic blood is needed	0.09908	0.0584038	0.090	−0.0153894	0.2135493
Current donor (yes)	**−0.2778558**	**0.0688061**	**0.000**	**−0.4127133**	**−0.1429982**
Social inclusion	**−0.3395684**	**0.1274423**	**0.008**	**−0.5893507**	**−0.089786**
Racial discrimination	0.0293386	0.1378402	0.831	−0.2408232	0.2995005
Family member has donated blood (yes)	**0.1535602**	**0.0539083**	**0.004**	**0.0479019**	**0.2592184**
Friends Has donated blood (yes)	0.0427983	0.0545869	0.433	−0.06419	0.1497867
Work colleague has donate blood (yes)	−0.0682494	0.0549802	0.214	−0.1760086	0.0395098
Neighbour has donated blood (yes)	0.0230505	0.0759652	0.762	−0.1258384	0.1719395
Constant	2.946199	0.2618483	0.000	2.432986	3.459413
*R* ^2^	0.29				

*Note*: Breusch–Pagan test of independence: *χ* ^2^(1) = 268.702, *p* = 0.0000 (*n* = 853).

*Note*: Figures in bold highlight the statistically significant effects. Coefficients in bold are all significant effects.

Abbreviations: CI, confidence interval; NHS, National Health Service; SE, standard deviation.

**TABLE 2:** Seemingly unrelated regression models for avoidance and negative word‐of‐mouth for travel abroad.

	Coefficient	Robust	*p* value	95% CI	
		SE		Lower	Upper
Outcome: avoidance					
Gender male	0.0786634	0.0497895	0.114	−0.0189222	0.1762489
Age	0.0014635	0.001945	0.452	−0.0023487	0.0052757
Need to explain	**0.0548282**	**0.0169459**	**0.001**	**0.0216148**	**0.0880415**
The question is to ensure safe blood	**−0.6196312**	**0.0485242**	**0.000**	**−0.7147368**	**−0.5245255**
Ethnicity (comparison is White)					
Mixed	0.0709655	0.0608869	0.244	−0.0483705	0.1903016
Asian	0.047944	0.0680526	0.481	−0.0854367	0.1813247
Black	**0.2804929**	**0.084801**	**0.001**	**0.114286**	**0.4466998**
Not aware that ethnic blood is needed	0.0368851	0.049103	0.453	−0.0593549	0.1331251
Current donor (yes)	**−0.2600327**	**0.0676046**	**0.000**	**−0.3925352**	**−0.1275302**
Social inclusion	−0.1897651	0.1242415	0.127	−0.433274	0.0537437
Racial discrimination within NHS	**0.2751077**	**0.1308768**	**0.036**	**0.0185938**	**0.5316216**
Family member has donated blood (yes)	0.0475548	0.0465199	0.307	−0.0436225	0.1387322
Friends has donated blood (yes)	0.0210827	0.0508747	0.679	−0.0786298	0.1207952
Work colleague has donate blood (yes)	−0.0634191	0.0459313	0.167	−0.1534428	0.0266046
Neighbour has donated blood (yes)	−0.0235403	0.0545463	0.666	−0.1304491	0.0833684
Constant	**4.229541**	**0.291407**	**0.000**	**3.658394**	**4.800689**
*R* ^2^	0.37				
Outcome: negative word‐of‐mouth					
Gender (male)	0.0334401	0.0461587	0.469	−0.0570293	0.1239096
Age	**0.0056782**	**0.0019385**	**0.003**	**0.0018787**	**0.0094777**
Need to explain	0.0091406	0.017519	0.602	−0.0251961	0.0434773
The question is to ensure safe blood	**−0.5002299**	**−0.0543796**	**0.000**	**−0.6068119**	**−0.3936478**
Ethnicity (comparison is White)					
Mixed	0.0704963	0.054624	0.197	−0.0365648	0.1775574
Asian	**0.1490424**	**0.0601033**	**0.013**	**0.0312421**	**0.2668427**
Black	**0.4367506**	**0.0924292**	**0.000**	**0.2555927**	**0.6179085**
Not aware that ethnic blood is needed	0.0125074	0.0438354	0.775	−0.0734085	0.0984233
Current donor (yes)	**−0.1644094**	**0.0649275**	**0.011**	**−0.2916649**	**−0.0371538**
Social inclusion	−0.1327164	0.1149905	0.248	−0.3580936	0.0926608
Racial discrimination within NHS	0.2428332	0.1325711	0.067	−0.0170014	0.5026677
Family member has donated blood (yes)	**0.0892877**	**0.0451744**	**0.048**	**0.0007475**	**0.1778278**
Friends has donated blood (yes)	−0.0844568	0.0470661	0.073	−0.1767045	0.007791
Work colleague has donate blood (yes)	−0.0249093	0.0441789	0.573	−0.1114984	0.0616798
Neighbour has donated blood (yes)	0.047215	0.0566966	0.405	−0.0639082	0.1583383
Constant	3.495889	0.2959177	0.000	2.915901	4.075877
*R* ^2^	0.31				

*Note*: Breusch–Pagan test of independence: *χ* ^2^(1) = 277.549, *p* = 0.0000 (*n* = 850).

*Note*: Figures in bold highlight the statistically significant effects. Coefficients in bold are all significant effects.

Abbreviations: CI, confidence interval; NHS, National Health Service; SE, standard deviation.

**FIGURE 2:**
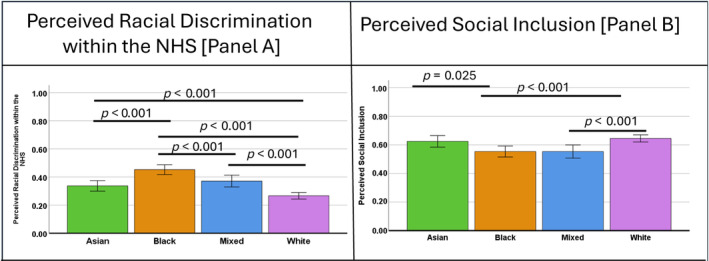
Levels of perceived racial discrimination within the NHS (Panel A) and social inclusion (Panel B). Panel (A) reports levels of perceived racial discrimination in the NHS on a standardized 0–1 scale where 1 is 100% complete discrimination and 0 is little to none. Panel (B) reports on perceived social inclusion on a standardized 0–1 scale where 1 is 100% inclusion 0 is little to none (exclusion). For Panel (A), the non‐overlapping 95% confidence interval (CI) indicates that all ethnic groups are significantly different from each other in terms of perceived racial discrimination in the NHS (except Asian and Mixed group). For Panel (B), the pattern of the 95% CI indicates that people from Black and Mixed ethnicities are not significantly different from each other, nor are Asian and White people, but both Asian and White people are significantly different from Black and also White people are significantly different from people with Mixed ethnicity. Differences in Panel (A) and (B) are reported using Bonferroni post hoc tests.

Supporting H2, Figure 2 shows that perceived discrimination within the NHS is higher in all ethnic minorities compared with White people and higher in Black people than people of Asian and Mixed ethnicities. Social inclusion is lowered in people of Black and Mixed ethnicities compared to White people, with social inclusion lower in Black compared with Asian people.

Hypothesis 3a,b Mediation by perceived racial discrimination and social isolation.

**FIGURE 3:**
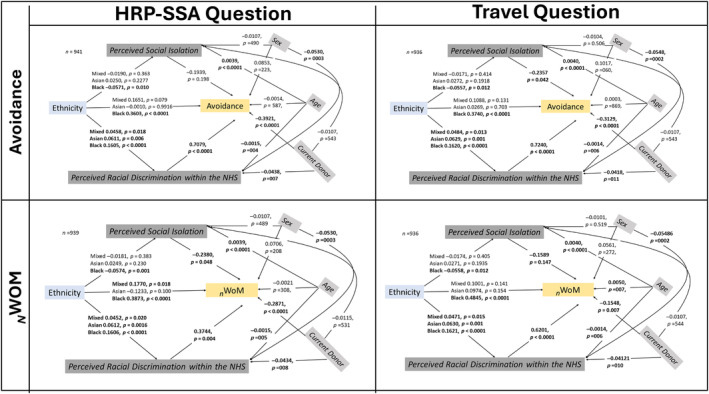
Parallel mediation models for ethnicity on avoidance and negative feedback via perceived ethnicity: White = comparison; sex (0 = female, 1 = male), current donor (0 = non‐donors, 1 = current donor). HRP‐SSA, higher risk partner from sub‐Saharan Africa question; _*N*_WoM, negative word‐of‐mouth.

Figure 3 shows the parallel mediation models for avoidance and _*N*_WoM. Consistent with H3a, for people from ethnic minorities, there was a significant indirect effect on both avoidance and _*N*_WoM via perceptions of higher racial discrimination. H3b was not supported as there was no indirect effect via social inclusion (Supplementary File S7 for more details).

Hypothesis 4a–c Ameliorates effects of awareness.

**FIGURE 4:**
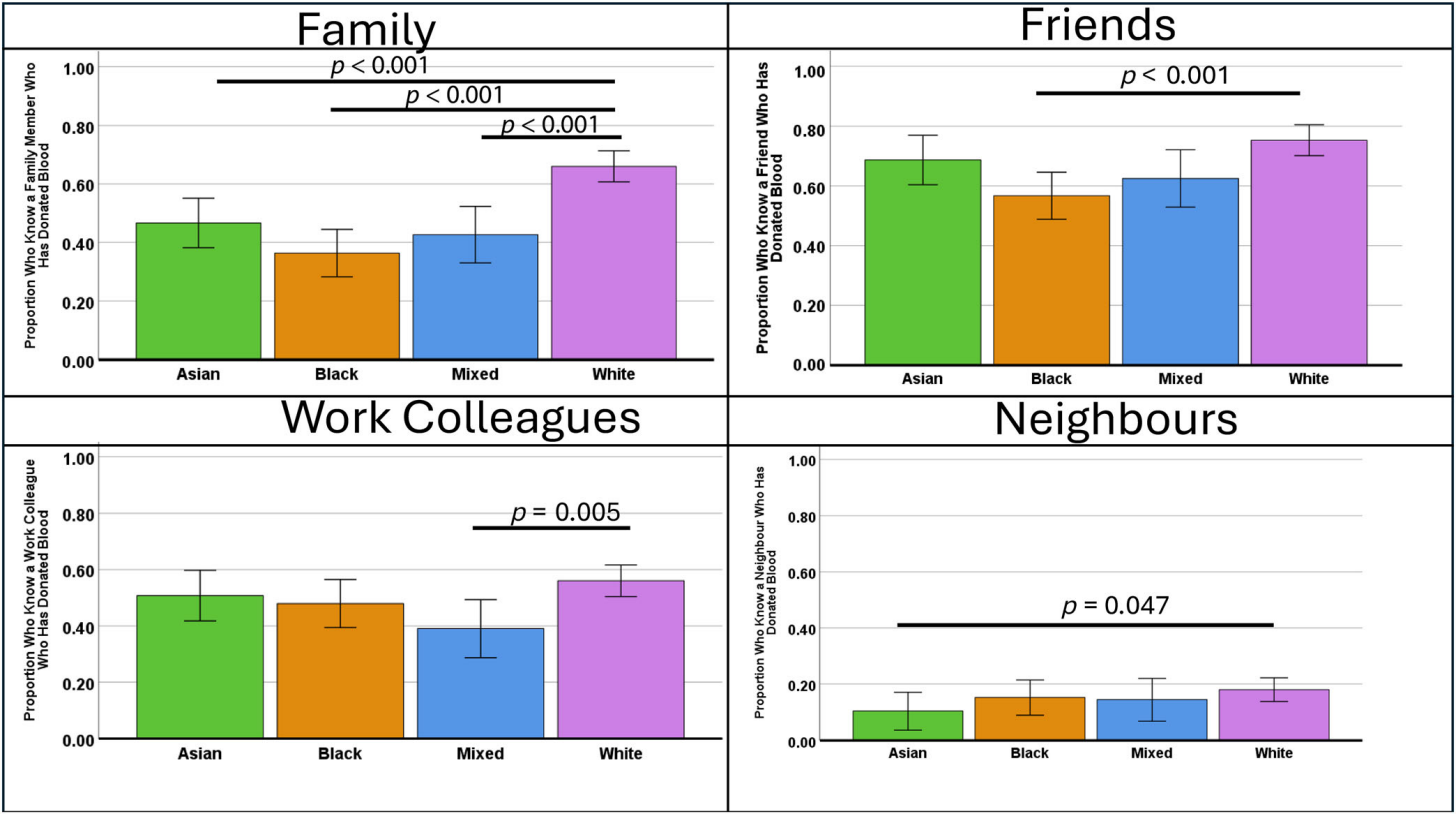
Percentage of family, friends, work colleagues and neighbours who are blood donors by ethnicity.

Figure 4 shows that family, friends and colleagues are more likely to be blood donors than neighbours. For White people, compared with all ethnic communities, family members are more likely to be blood donors. White people are also more likely to have friends as donors than Black people, work colleagues who donate compared with people of mixed ethnicity and neighbours who donate compared to Asian people.

Table 1 shows that avoidance was also *higher* if people (i) were less aware of the need for blood from ethnic minorities (supporting H4c) and (ii) knew a family member who has donated blood (not supporting H4a). Avoidance was *lower* if people believed the question was included to ensure safety (supporting H4b). _*N*_WoM was *higher* if people (i) knew a family member who had donated blood (not supporting H4a) and lower if they believed the question was included to ensure safety of blood (supporting H4b). For the Travel question (Table 2) both avoidance and _*N*_WOM were lower when people believed the question was included to ensure blood safety (supporting H4b), and _*N*_WOM was higher if people knew a family member who had donated blood (not suppressing H4a).

There were no moderating effects of knowing a family member who donates (see Supplementary files S8–S10 for details). However, there is a significant interaction between ethnicity and donor status on _*N*_WoM, such that Black and Mixed current donors are less likely to tell others not to donate than Black and Mixed non‐donors (see Supplementary File S8; Figure S1).

## STUDY 2: EPIDEMIOLOGY OF HRP‐SSA ON HIV RATES AND TRAVEL QUESTIONS ON DEFERRAL

### Methods

All UK donations are tested for markers of HIV and other blood‐borne viruses. Based on this routine surveillance for the four UK blood services, data for each donor with confirmed HIV identified through donation screening in the United Kingdom from 1996 to 2019 were extracted from the joint NHSBT/UK Health Security Agency (UKHSA) Epidemiology Unit database [29]. Data fields included the confirmatory testing results, index donation date, most recent previous donation, gender, age, ethnicity, country of birth, probable exposure route and compliance. Data on partners giving samples to NHSBT and deferral data from donation sessions were provided on a one‐off basis by each blood service where available from their donor management system. Annual data on malaria deferrals advised by the National Call Centre between 2015 and 2019 were provided by ethnic background and compared with annual data on donors making whole blood donations, calculating the deferrals as a percentage of donations made by each ethnic background (as in [29]).

### Results

The detailed results are in Table 3. The proportion of UK donors with HIV attributed to HRP‐SSA has decreased over time, with HRP‐SSA being assigned as the possible exposure in 24% of donors with HIV between 1996 and 2019 and 10% (5/49) of donors with HIV between 2015 and 2019. Looking at recent HIV acquired within 12 months, 14% (19/132) reported HRP‐SSA for 1996–2019 and 8% (1/12) for 2015–2019. Of these 19 donors with recent HIV, 6 reported a regular partner who may have had sex in Africa as their only risk. Of the 132 donors with recent HIV, 6 were detected in the window period i.e. HIV antibody negative, RNA positive, indicating that HIV was acquired extremely recently. Three of these reported HRP‐SSA, including one with another possible exposure, the most recent in 2008. (Supplementary File S11 for additional data). The number of partners of potential donors who gave samples, allowing their partner to donate, was small, 60 in England in 2020, but none were found to be living with HIV. Other countries within the UK deferred without an option for partner testing. There were around 50 and 16 deferrals on session annually in Scotland and Wales, respectively.

Hypothesis 5 People from ethnic minorities were more likely to be deferred after travel.

**TABLE 3:** HIV in blood donors, all and recent infection, UK 2015–2019.

	HIV all	HIV recent	% which are recent	% of recent infections
Total	49	12	24.5	
NAT pick up	‐	1		
Seroconversion	‐	10		
Gender				
Male	31	11	35.5	91.7
Female	18	1	5.6	8.3
Donor type				
New	24	2	8.3	16.7
Repeat	25	10	40.0	83.3
Age				0.0
Age‐range	18–71	28–60		
Median age	37	42.5		
Ethnicity				
Asian	5	1	20.0	8.3
Black	2	0	0.0	0.0
Not known	1	0	0.0	0.0
Other	2	0	0.0	0.0
White	39	11	28.2	91.7
Born				
United Kingdom	31	4	12.9	33.3
Europe	6	2	33.3	16.7
Asia	2	1	50.0	8.3
Africa	1	0	0.0	0.0
Other	2	0	0.0	0.0
Not known	7	5	71.4	41.7
Acquired infection				
United Kingdom	29	8	27.6	66.7
Europe	5	2	40.0	16.7
Asia	3	1	33.3	8.3
Africa	1	0	0.0	0.0
Other	0	0	‐	
Not known	9	1	11.1	8.3
Risk group				
GBM	13	6	46.2	50.0
Heterosexual sex	23	3	13.6	25.0
HRP‐SSA	5	1	20.0	8.3
HRP‐other	3	1	20.0	8.3
Other	1	0		0.0
Not known	4	1	25.0	8.3

Abbreviations: HIV, human immunodeficiency virus; HRP‐SSA, higher risk partner from sub‐Saharan Africa; GBM, gay and bisexual men; NAT, nucleic acid test.

From 2015 to 2019, the average percentage of Asian‐Indian people who were advised not to donate out of those making a donation was 8.2% (range = 6.3%–14.9%), Asian‐Pakistani 9.3% (range = 6.8%–12.3%), Black‐African 8.0% (range = 5.2%–9.9%) and White 1.7% (range = 1.5%–2.0%). These are similar to the figures recorded in 2015 when the malaria deferral was six months [25].

## DISCUSSION

The results are clear: those from ethnic minorities are more likely to be put off donating and discourage others after reading the HRP‐SSA and travel questions used in the United Kingdom in 2019. These effects were mediated through perceived racial discrimination within the NHS. Thus, there are clear negative behavioural effects associated with these 2019 questions. The epidemiology data showed that the HRP‐SSA question was linked to a small proportion of HIV+ donations and was part of a downward trend. Thus, the combined behavioural and epidemiology data indicated that the removal of the HRP‐SSA question was justified and safe, and indeed based, in part, on these data, this question was removed from Scotland, Wales, Northern Ireland and England in 2021 as part of the FAIR project (https://www.blood.co.uk/news-and-campaigns/news-and-statements/fair-steering-group/).

### The spread of avoidance and _*N*_WoM

The impact of avoidance and _*N*_WoM in the community can spread quickly. Avoidance can result in the lone‐wolf effect, whereby observing others choosing not to act sends a social signal that not donating is preferred [30]. _*N*_WoM also spreads quickly through communities [8]. Thus, the summative effect of the lone‐wolf effect and _*N*_WoM, reinforced by perceived racial discrimination within the NHS, creates a complex social milieu for recruitment.

We initially hypothesized that knowing a family member who donates would mitigate these negative effects. However, we observe the opposite: knowing family members directly enhances the negative impact. There are several possibilities for this. First, people may feel these questions are unjustified and, as such, are upset on behalf of their families. Second, as they already know others who donate, they may feel less need to donate or encourage others. Third, they may know a family member who has been deferred.

### Practical and clinical implications

In terms of the Travel question, malaria antibody testing was introduced consistently in England from 2001 as a way of reducing the deferral burden on Black and Asian donors. This deferral and testing strategy has been reviewed and reduced to the shortest deferral time that is thought safely possible under the current antibody testing strategy at 4 months post‐travel [31]. In terms of people calling to check eligibility on the grounds of travel before donation, the NHSBT National Call Centre data showed that between 2015 and 2019, Asian and Black donors were more likely to be advised not to donate due to travel than White donors, with figures consistent with a previous observation made under 6‐month deferral [25].

Providing a rationale for the pre‐donation questions can reduce their negative impact. Thus, blood services must provide clear information about why these questions are needed and that they only remain if the evidence supports them. Each UK blood service explains on its website that the questions are to keep recipients and donors safe. Further work is underway to simplify the travel questions and study ways to prompt donors to disclose relevant history. Services may encourage _*P*_WoM as a potential to counter‐act _*N*_WoM; however, people acting altruistically are reluctant to use _*P*_WoM publicly [16], and it may not be effective anyway (see [12]). An optimal strategy, however, may be to intervene earlier downstream to create a positive experience for all donors, not just in terms of the social ambience of the centres and staff but more structurally in terms of how and when deferral questions are asked, what is asked and the ethnicity of staff.

Finally, perceived racial discrimination within the NHS was an important mechanism supporting _*N*_WoM in ethnic minorities. It is beyond the capacity of blood services to address this wider socio‐political issue. However, this discrimination should be recognized and publicly acknowledged in terms of openness and transparency.

### Caveats

As these findings are UK‐specific, generalizability should not be assumed. Blood services with similar questions should evaluate them for similar negative impacts, considering the appropriate local HIV epidemiology and the behavioural impact. We did not assess donor knowledge and attitudes, and further work should explore how these influence _*P*_WoM as a function of ethnicity and other demographics. Finally, it should also be noted that the number of non‐donors is small.

## CONFLICT OF INTEREST STATEMENT

The authors declare no conflicts of interest.

## Supporting information


**Data S1:** Supporting Information.

## Data Availability

Data reported in this paper are available from the first author on request.

## References

[1] Shaz BH , Zimring JC , Demmons DG , Hillyer CD . Blood donation and blood transfusion: special considerations for African Americans. Transfus Med Rev. 2008;22:202–214. 18572096 10.1016/j.tmrv.2008.02.006

[2] Mwamba N , Puplampu KP . Blood, it's in you to give, just don't be an African: the Canadian blood system and the African Indefinite Deferral Policy, 1997 to 2018. Identities. 2023;31:1–19.

[3] Halperin D , Baetens J , Newman B . The effect of short‐term, temporary deferral on future blood donation. Transfusion. 1998;38:181–183. 9531951 10.1046/j.1537-2995.1998.38298193102.x

[4] Ribeiro DA , Kalro AD . Four decades of negative word‐of‐mouth and negative electronic word‐of‐mouth: a morphological analysis. Int J Consum Stud. 2023;47:2528–2552.

[5] Rosario AB , Sotgiu F , De Valck K , Bijmolt THA . The effect of electronic word of mouth sales: a meta‐analytic review of platform, product, and metric factors. J Market Res. 2016;53:297–318.

[6] Martin S , Greiling D , Leibetseder N . Effects of word‐of‐mouth on the behavior of Austrian blood donors: a case study of the red cross blood donation service. Health Promot Int. 2019;34:429–439. 29253143 10.1093/heapro/dax086

[7] Ito TA , Larsen JT , Smith NK , Cacioppo JT . Negative information weighs more heavily on the brain: the negativity bias in evaluative categorisations. J Pers Soc Psychol. 1998;75:887–900. 9825526 10.1037//0022-3514.75.4.887

[8] Johnson NF , Velásquez N , Restrepo NJ , Leahy R , Gabriel N , El Oud S , et al. The online competition between pro‐ and anti‐vaccination views. Nature. 2020;582:230–233. 32499650 10.1038/s41586-020-2281-1

[9] Fay N , Walker B , Kashima Y , Perfors A . Socially situated transmission: the bias to transmit negative information is moderated by the social context. Cognit Sci. 2021;45:e13033. 34490917 10.1111/cogs.13033

[10] Arruda Filho EJM , Barcelos ADA . Negative online word‐of‐mouth: Consumers' retaliation in the digital world. J Glob Mark. 2020;2020:19–37.

[11] Feng Y , Du L , Ling Q . How social media strategies of nonprofit organizations affect consumer donation intention and word‐of‐mouth. Soc Behav Pers. 2017;45:1775–1786.

[12] Schröder JM , Merz EM , Suanet B , Wiepking P . Did you donate? Talking about donations predicts compliance with solicitations for donations. PLoS One. 2023;18:e0281214. 36730274 10.1371/journal.pone.0281214 PMC9894400

[13] Peedin AR , Park YA , Mazepa MA , Siniard RC , Neish T , Raval JS . The impact of an undergraduate biology class on donor recruitment at a hospital‐based blood donor center. Am J Clin Pathol. 2020;153:368–373. 31783402 10.1093/ajcp/aqz177

[14] Sundermann LM . Share experiences: receiving word of mouth and its effect on relationships with donors. J Serv Mark. 2018;32:322–333.

[15] Rahay I , Hermawan H , Ekasari A . Electronic effects of word of mouth and perceived value on blood donor intention: theory of planned behavior. Int J Humanit Educ Soc Sci. 2023;3:3.

[16] Previte J , Russell‐Bennett R , Mulcahy R , Hartel C . The role of emotional value for reading and giving eWoM in altruistic services. J Bus Res. 2019;99:157–166.

[17] Ramondt S , Kerkhof P , Merz EM . Blood donation narratives on social media: A topic modeling study. Transfus Med Rev. 2022;36:58–65. 34810071 10.1016/j.tmrv.2021.10.001

[18] Ferguson E , Dawe‐Lane E , Khan Z , Reynolds C , Davison K , Edge D , et al. Trust and distrust: Identifying recruitment targets for ethnic minority blood donors. Transfus Med. 2022;32:276–287. 35499471 10.1111/tme.12867 PMC9542243

[19] Hamed S , Bradby H , Ahlberg BM , Thapar‐Björkert S . Racism in healthcare: a scoping review. BMC Public Health. 2022;22:988. 35578322 10.1186/s12889-022-13122-y PMC9112453

[20] Klinkenberg EF , Huis In 't Veld EMJ , de Wit PD , de Kort WLAM , Fransen MP . Barriers and motivators of Ghanaian and African‐Surinamese migrants to donate blood. Health Soc Care Community. 2019;27:748–756. 30478863 10.1111/hsc.12692 PMC7379538

[21] Polonsky MJ , Brijnath B , Renzaho AM . "They don't want our blood": social inclusion and blood donation among African migrants in Australia. Soc Sci Med. 2011;73:336–342. 21704441 10.1016/j.socscimed.2011.05.030

[22] Scheidmeir M , Kubiak T , Luszczynsk A , Wendt J , Scheller DA , Meshkovska B , et al. Acceptability of policies targeting dietary behaviours and physical activity: a systematic review of tools and outcomes. Euro J Pub Health. 2022;32:iv32–iv49. 10.1093/eurpub/ckac053 PMC9897019 36444105

[23] Beerli‐Palacio A , Martín‐Santana JD . Model explaining the predisposition to donate blood from the social marketing perspective. Int J Nonprofit Volunt Sect Mark. 2009;14:205–214.

[24] Ferguson E , Hill A , Lam M , Reynolds C , Davison K , Lawrence C , et al. Typology of blood donor motivations. Transfusion. 2020;60:2010–2020. 32618010 10.1111/trf.15913

[25] Reynolds C , Cieply L , Sell J , Brailsford SR . Who do we gain? Enhancement of blood supplies by additional testing for donors who travel. Transfus Med. 2019;29:325–331. 31347219 10.1111/tme.12620

[26] LaVeist TA , Nickerson KJ , Bowie JV . Attitudes about racism, medical mistrust, and satisfaction with care among African American and white cardiac patients. Med Care Res Rev. 2000;57:146–161. 11092161 10.1177/1077558700057001S07

[27] Huxley P , Evans S , Madge S , Webber M , Burchardt T , McDaid D , et al. Development of a social inclusion index to capture subjective and objective life domains (phase II): psychometric development study. Health Technol Assess. 2012;16:iii–241. 10.3310/hta16010 22260923

[28] U.K. Department for Communities and Local Government . Citizenship survey (2010–2011). Neighbourhood and community life survey (2020–21). Available from: https://www.gov.uk/government/statistics/community‐life‐survey‐202021‐neighbourhood‐and‐community/neighbourhood‐and‐community‐community‐life‐survey‐202021#:~:text=Respondents%20were%20asked%20how%20strongly,2019%2F20%20(63%25). Last accessed 8 Apr 2024.

[29] Reynolds C , Davison KL , Brailsford SR . Safe supplies: few infections in UK blood and tissue donors. Transfus Med. 2019;29:239–246. 30689250 10.1111/tme.12576

[30] Ferguson E , Shichma R , Tan JH . Lone wolf defectors undermine the power of the opt‐out default. Sci Res. 2020;10:8973. 10.1038/s41598-020-65163-1 PMC7265288 32488105

[31] Kitchen AD , Lowe PH , Lalloo K , Chiodini PL . Evaluation of a malarial antibody assay for use in the screening of blood and tissue products for clinical use. Vox Sang. 2004;87:150–155. 15569066 10.1111/j.1423-0410.2004.00561.x

