# Supplementary material for: Questions on travel and sexual behaviours negatively impact ethnic minority donor recruitment: Effect of negative word‐of‐mouth and avoidance
Source: Vox Sang. 2024 Nov 6;119(12):1245–56. doi: 10.1111/vox.13748 (PMC11634444; doi:10.1111/vox.13748)
Supplement: Supplementary file 1 — Data S1: Supporting Information. [file VOX-119-1245-s001.docx]

**Supplementary Files for: Questions on Travels and Sexual Behaviours Negative Impact on Ethnic Minority Donor Recruitment: Effects of Negative Word-of-Mouth and Avoidance**

Table of Contents

[Supplementary Files S1: Sampling procedure 3](#_Toc171084536)

[**Sampling Procedures** 3](#_Toc171084537)

[Supplementary File S2: ONS Categorization 5](#_Toc171084538)

[***Table S1***. *Coding of Self-Defined Ethnicity* 5](#_Toc171084539)

[***Table S2***. *Sample Characteristics* 6](#_Toc171084540)

[Supplementary File S3: Wider Structure of the Survey 7](#_Toc171084541)

[Supplementary File S4: Family/Friend as Donord, Racial Discrimination, Social Isolation. HRP-SSA and Travel Question Measures 8](#_Toc171084542)

[**Knowing Family and Friends who have donated blood.** 8](#_Toc171084543)

[**Racial Discrimination within the NHS** 8](#_Toc171084544)

[**Social Inclusion** 8](#_Toc171084545)

[**HRP-SSA Question and Assessment** 9](#_Toc171084546)

[**Travel Question and Assessment** 9](#_Toc171084547)

[Supplementary File S5: Standardizing Scores and Power Calculations 10](#_Toc171084548)

[**Standardization of Scores** 10](#_Toc171084549)

[**Power Calculations** 10](#_Toc171084550)

[Supplementary File S6: Percentages Agree, Neutral, and Disgaree by Ethnicity and Donor Status 12](#_Toc171084551)

[***Table S3***. *Percentage Agreement and Disagree with Avoidance and _N_WOM.* 13](#_Toc171084552)

[Supplementary File S7: Mediation Coefficients 14](#_Toc171084553)

[***Table S4***. Indirect Effects of Ethnicity on Avoidance and _N_WOM via Perceived Racial Discrimination (M_1_] and Perceived Social Inclusion [M_2_] 15](#_Toc171084554)

[Supplementary File S8: Moderation of Ethnicity Effects on Avoidance and Negative Feedback by Donor Status 16](#_Toc171084555)

[***Table S5*** *Moderation of Ethnicity on Avoidance and Negative Feedback by Donor Status for Having Sex with Someone who may have had sex with someone in an HIV-Endemic area* 16](#_Toc171084556)

[***Table S6***. *Moderation of Ethnicity on Avoidance and _N_WOM by Donor Status for Travel to a Malarious Area* 18](#_Toc171084557)

[Supplementary File S9: Moderation by Family Member Being a Donor on Travel with Respect to Donor Status 19](#_Toc171084558)

[***Non-Current Donors Travel*** 19](#_Toc171084559)

[***Table S7*** *Moderation of Ethnicity on Avoidance and _N_WOM by Donor Status for Travel to a Malarious Area (Non-Donors)* 19](#_Toc171084560)

[***Current-Donors Travel*** 20](#_Toc171084561)

[***Table S8***. *Moderation of Ethnicity on Avoidance and _N_WOM* *by Donor Status for Travel to a Malarious Area (Current Donors)* 20](#_Toc171084562)

[Supplementary File S10: Moderation by Family Member Being a Donor on Sex with Respect to Donor Status 21](#_Toc171084563)

[***Non-Current Donors Sex*** 21](#_Toc171084564)

[***Table S9***. *Moderation of Ethnicity on Avoidance and _N_WOM by Donor Status for Sex (Non-Donors)* 21](#_Toc171084565)

[***Current-Donors Sex*** 22](#_Toc171084566)

[***Table S10***. *Moderation of Ethnicity on Avoidance and _N_WOM by Donor Status for Sex (Current Donors)* 22](#_Toc171084567)

[Supplementary Files S11: Epidemiology Tables 23](#_Toc171084568)

[***Table S11:*** ***Number of recently acquired infections (<12m) in blood donors, looking at area sexually acquired, UK 2015-2019*** 23](#_Toc171084569)

# Supplementary Files S1: Sampling procedure

This section details the sampling procedures.

## **Sampling Procedures**

**NHSBT:** ethnic minorities

A random sample of 3,500 ethnic minorities (including White minorities) and 2,500 donors from White backgrounds were recruited, as long as they had not opted out of NHSBT communications or had medical deferrals and had not been sampled in other NHSBT research/surveys in the last 6 months.

Participants were sampled if they: (1) were aged over 18, (2) had registered to donate blood and had donated in the last two years, as we are interested primarily in current active donors (this is how NHSBT defines a current active donor, those who have not donated for two years are archived as lapsed donors), (3) self-identified by their reported ethnic group as an ethnic minority (excluding White minorities) or people from a White background (including White minorities) based on self-reported PULSE codes (e..g., Caribbean, African, Any other Black/African/Caribbean background, MIXED White and Black Caribbean, White and Black African and English/Welsh/Scottish/Northern Irish/British, Irish, Gypsy or Irish Traveller) .and (4) had an e-mail address registered with NHSBT and not shared by 2 or more donors. Both male and female donors were sampled.

**Code 3:**

From the Code 3 database of 100,059 participants, a random sample of 4,300 ethnic minorities (excluding White minorities) (from 12,727) and 4,300 people from a White background (from 65,078) participants were sampled. Participants’ were sampled if they: (1) were aged over 18, (2) registered with Code 3 and had not withdrawn permission to be contacted, and (3) identified as ethnic minorities (excluding White minorities) (African or Caribbean, White/Black African and White/Black Caribbean, Indian and Pakistani and any other Asian background) or people from a White background (White British and White Irish and other White) group member.

**Community Group Booster Samples:**

We also recruited ethnic minority (excluding White minorities) people from several local community groups if they were: (1) aged over 18, (2) registered on the community groups email list who had given consent to be contacted by the community and had not withdrawn permission.

**Survey dates and reminders**

Initial surveys and reminders were sent on the 14^th^ of June 2019, with a reminder on the 12^th^ of July 2019. An additional reminder was sent to the ethnic minority sample on the 2^nd^ of August 2019

**Compensation**

NHSBT donors were not paid, and the 5 Code-3 donors were randomly selected to receive a £25 gift voucher.

# Supplementary File S2: ONS Categorization

Below are the ONC categorisation. The white sample does not include white minorities (e.g, Gypsy, Roma or Irish Traveller groups or specified as ‘White other’).

## ***Table S1***. *Coding of Self-Defined Ethnicity*

|  | n | Valid Percentage |
| --- | --- | --- |
|  |  |  |
| **People from Asian ethnic backgrounds** |  |  |
| Indian | 61 | 6.3% |
| Pakistani | 19 | 2.0% |
| Bangladeshi | 5 | 0.5% |
| Chinese | 1 | 0.1% |
| Any other Asian background, please describe | 96 | 10% |
|  |  |  |
| **People from Black and Caribbean backgrounds** |  |  |
| African | 35 | 3.6% |
| Caribbean | 32 | 3.3% |
| Any other Black/African/Caribbean background, please describe | 74 | 7.7% |
|  |  |  |
| **People from mixed ethnic backgrounds** |  |  |
| White and Black Caribbean | 39 | 4.1% |
| White and Black African | 10 | 1% |
| White and Asian | 37 | 3.9% |
| Any other Mixed/Multiple ethnic backgrounds, please describe | 69 | 7.2% |
| Black & White | 3 | 0.3% |
|  |  |  |
| **Other ethnic groups** |  |  |
| Arab | 8 | 0.8% |
| Any other ethnic group, please describe | 16 | 1.7% |
|  |  |  |
| **White People** |  |  |
| English/Welsh/Scottish/Northern Irish/British | 426 | 44.3% |
| Irish | 3 | 0.3% |
| Gypsy or Irish Traveller | 0 | 0% |
| Any other White background, please describe | 27 | 2.3% |
|  |  |  |
| Missing | 20 |  |

Any other White background included White/Anglo Saxon, White European, White Caucasian, White Jewish; Any other Mixed/Multiple ethnic backgrounds included ‘English, Caribbean, Indian’, ‘White British and African American’,’ White, Black Caribbean and Asian, ‘White and Guyanese (Indian-Caribbean)’, ‘white parent & mixed white/Caribbean parent’, ‘Anglo-Hispanic]; Any other Asian background included ‘Asian – Iranian, ‘Asian Japanese’, ‘British Indian – Punjabi’, ‘Burmese’, ‘Bengali’; Any other Black/African/Caribbean background included ‘Black British Caribbean’, ‘African American/Black’, ‘English & Jamaican’, ‘British Anglo Afro American’; Any other ethnic group included ‘Arab- mixed Black and White African, ‘Egyptian.’

## ***Table S2***. *Sample Characteristics*

|  |  | All | Non-Donors | Donors | Non-Donors vs Donors |
| --- | --- | --- | --- | --- | --- |
|  |  | n or Mean |  |  |  |
| NHSBT Donors | All ethnic minorities (excluding White minorities | 376 |  | 376 |  |
|  | White people | 343 |  | 343 |  |
| Code 3 (Market Research) | All ethnic minorities (excluding White minorities | 132 | 103 | 21 |  |
|  | White people | 122 | 111 | 19 |  |
| Community Group | People from an Asian background | 8 | 6 | 2 |  |
| Donor Status | Current Donors | 761 |  |  |  |
|  | Non-Donors | 220 |  |  |  |
| Ethnicity |  |  |  |  |  |
|  | Asian | 182 | 38 (17.3%) | 144 (19.4%) | χ^2^ _(3)_ = 24.43, p = .000. There were fewer donors from Black communities than expected. There were fewer non-donors from Asian communities than expected. |
|  | Black | 141 | 53 (24.1%) | 88 (11.9%) |  |
|  | Mixed | 182 | 27 (12.3%) | 155 (20.9%) |  |
|  | White | 456 | 102 (46.4%) | 354 (48.8%) |  |
|  | Missing Data | 20 |  |  |  |
| Sex | Male | 339 | 42 (19.3%) | 297(39.4%) | χ^2^ _(1)_ = 30.15, p = .000. There were more male donors than expected, and fewer female non-donors than expected |
|  | Female  Missing Data | 633  9 | 176 (89.7%)  2 | 457 (60.6%)  7 |  |
|  |  |  |  |  |  |
| Age |  | *M* = 44.65 (SD = 14.57) Range 18-89 | *M* = 46.05 (SD = 14.15) | *M* = 44.23 (SD = 14.67) | t (963) = 1.63, p = .193 |

***Note***. Current Donors = donated within the last 2 years. Asian = People from Asian ethnic backgrounds, Black = People from Black and Caribbean backgrounds; Mixed = People from mixed ethnic backgrounds, White = White People (excluding White minorities)

# Supplementary File S3: Wider Structure of the Survey

The survey had the following sections.

| **Area** | **Focus** |
| --- | --- |
| **Demographics** | Age, sex, self-defined ethnicity, |
| **Previous Donor Status** | Have you ever donated blood? Yes No  If yes – was this in the UK or abroad? UK Abroad  If yes – when was the last time you donated? Less than a month ago, 2 to 12 months ago, 12 months to 2 years ago, Longer than 2 years ago, Can’t remember. |
| **Family Donate Blood** | Do you know any people from the following groups who have donated blood? Your family, your friends, your work colleagues, your neighbours |
| **Willingness to Donate** | Would you consider donating blood in the future?  Yes No |
| **Awareness** | Awareness of the need for BAME blood and awareness of NHSBT campaigns |
| **Trust** | Questions derived to assess trust in (1) NHS and UK health care, (2) NHSBT, (3) other groups (nationalities, religions), (4) organisations (police, courts, government), and (5) strangers and (7) items measuring distrust based on ethnicity and social class (see main text for details) |
| **Altruism** | We asked participants to respond to 4 hypothetical dictator games (DGs) to assess altruism. For each DG, we asked participants to split £50 between themselves and (1) a relative, (2) a stranger, (3) an overseas charity, and (4) a local community event. |
| **Social Inclusion** | We asked about social isolation within the participant’s local community and the UK in general. |
| **Racial Discrimination** | We asked about racial discrimination within the health service specifically and in general. |
| **Donor Health Check** | We asked participants to consider and evaluate three UK Donor Health Check (DHC) questions. These questions focused on (1) having sex with someone from areas with a high prevalence of HIV/AIDS, (2) returning from an area where there is malaria, including many parts of Africa, Asia, and South America in the last four months they are asked not to donate and (3) if they were born or ever lived or stayed outside the UK for a continuous period of 6 months or more. |
| **Socio-Political Context** | We asked whether people perceived Brexit as a positive move for the UK, whether they were aware of the Windrush Scandal, and to what extent participants perceived it indicates that the UK Government has a negative view of ethnic minorities. |

# Supplementary File S4: Family/Friend as Donord, Racial Discrimination, Social Isolation. HRP-SSA and Travel Question Measures

## **Knowing Family and Friends who have donated blood.**

| Do you know any people from the following groups who have donated blood? | | | |
| --- | --- | --- | --- |
| Your family | Yes | No | Don’t know |
| Your friends | Yes | No | Don’t know |
| Your work colleagues | Yes | No | Don’t know |
| Your neighborhood | Yes | No | Don’t know |

## **Racial Discrimination within the NHS**

**Racial Discrimination with the NHS**: People were asked their levels of (dis)agreement with the following three items from LaVeist, Nickerson & Bowie (2000): (i) Racial discrimination in a doctor’s surgery is common, (ii) Patients get the same medical treatment from the National Health Service (NHS) no matter what the patient’s race or ethnicity (reverse scored), and (iii) People of my ethnic group are treated the same as people of other groups by doctors and healthcare workers (reverse scored),. These were responded to on a 5-point scale (1 = Strongly Disagree, 2 = Disagree, 3 = Neither Disagree or Agree, 4 = Agree and 5 = Strongly Agree). Items 2 and 3 were reverse scores, and the three items totalled to give a single scale of racial discrimination within the NHS such that higher scores equated to greater discrimination (α = .84, *M* = 6.75, *SD* = 2.58).

## **Social Inclusion**

**Social Inclusion**: People were asked the following two items: (i) How strongly do you feel you belong to your immediate community/neighbourhood, and (ii) Overall, how strongly do you feel about the extent to which you are included in broader society in the UK, which were responded to on a 5-point scale (1 = Don’t know, 2 = Not at all Strongly, 3 = Not Very Strongly, 4 = Fairly Strongly and 5 = Strongly Agree) (Huxley P, Evans S, Madge S, et al., 2012; U.K. Department for Communities and Local Government, 2021). Items were totalled to give a single scale of social inclusion such that higher scores equated to greater social inclusion (α = .73, *M* = 6.87, *SD* = 1.82)

## **HRP-SSA Question and Assessment**

Before donating blood, everyone must read an information booklet and complete a form that asks questions about lifestyle, health, and travel. In one question, those presenting to donate blood are asked if, in the last 3 months, they have *“had sex with anyone who may ever have had sex in parts of the world where AIDS/HIV is very common (this includes most countries in Africa)?”* If they answer Yes they are asked not to donate unless their partner can give a sample for testing.

Please think about this question in general and answer the following questions:

| *This question:* | Strongly Disagree | Disagree | **Neither disagree nor agree** | Agree | Strongly agree |
| --- | --- | --- | --- | --- | --- |
| Needs to be asked to keep blood safe for patients | 1 | 2 | 3 | 4 | 5 |
| Would put me off wanting to donate blood | 1 | 2 | 3 | 4 | 5 |
| Makes me want to tell others not to donate | 1 | 2 | 3 | 4 | 5 |
| The reason for asking this needs to be explained to the donor | 1 | 2 | 3 | 4 | 5 |

## **Travel Question and Assessment**

Before donating blood, everyone must read an information booklet and complete a form that asks questions about lifestyle, health, and travel. In one question, those presenting to donate blood are asked if they have travelled outside the UK in the last 12 months or since their last donation. Specifically, if donors have returned from an area with malaria, including many parts of Africa, Asia and South America in the last 4 months, they are asked not to donate.

Please think about this question in general and answer the following questions:

| *This question:* | Strongly Disagree | Disagree | **Neither disagree nor agree** | Agree | Strongly agree |
| --- | --- | --- | --- | --- | --- |
| Needs to be asked to keep blood safe for patients | 1 | 2 | 3 | 4 | 5 |
| Would put me off wanting to donate blood | 1 | 2 | 3 | 4 | 5 |
| Makes me want to tell others not to donate | 1 | 2 | 3 | 4 | 5 |
| The reason for asking this needs to be explained to the donor | 1 | 2 | 3 | 4 | 5 |

# Supplementary File S5: Standardizing Scores and Power Calculations

## **Standardization of Scores**

Trust scores were standardised using the following linear transform:

$$normalize{d score}_{i}=\frac{score_{i}-minscore}{maxscore-minscore}$$

Thus, a score of 0 equates to no trust at all or complete lack of distrust, and 1 equates to complete trust or distrust. Scores in between these values indicate the degree of trust or distrust based on the re-scaled range (*max-min*). As this is a standard linear transform, it does not alter the relationship between predictors and outcomes; rather, it aids interpretation.

## **Power Calculations**

Power calculations were conducted to achieve .80 power with an α of .05 (two-tailed). As no existing data on avoidance and negative feedback following DHC questions exist, calculations are based on variation in conditional distrust in the health services by ethnicity reported by Ferguson et al. (2022), which has a Cohen’s d of 0.5231. This indicated that for comparison across 4 groups (Asian, Black, Mixed, White), 58 per group are needed. Regarding perceived racial discrimination, the average effect size comparing Asian, Black, and Mixed ethnicities to White is a Cohen’s D of 0.34 (Shariff-Marco, Breen N, Landrine H, et al., 2011), equating to 136 per group. For social inclusion, the average effect size comparing Asian, Black, and Mixed ethnicities to White is a Cohen’s D of 0.285, equating to 193 per group (Dykxhoorn, Osborn, Fischer, Troy, Kirkbride & Walters, 2023). These effects are stronger for Black and Mixed ethnicities compared to Asian people (Dykxhoorn et al., 2023; Shariff-Marco et al., .2011). For example, for Black vs White people, the effect size for racial discrimination is Cohen’s d of 0.42, requiring 90 people per group (Shariff-Marco et al. .2011). For social inclusion, Cohen’s D is 0.414, requiring 93 people per group (Dykxhoorn et al., 2023). Power calculations for a parallel mediation model (M_1_ = racial discrimination, M_2_ = social isolation) derived using methods developed by Schoemann, Boulton, and Short (2017), and were based on the effect sizes above, with the M1 and M2 to Y associations specified as small. Based on a simulation with 1000 repetitions and 20,000 Monte Carlo draws per repetition, it indicates that the indirect path from ethnicity via racial discrimination (M1) requires 500 people, and the indirect path from ethnicity via social inclusion (M_2_) requires 560 people.

**References**

Dykxhoorn J, Osborn D, Fischer L, Troy D, Kirkbride JB, Walters K. (2023). Measuring social exclusion and its distribution in England. *Soc Psychiatry Psychiatr Epidemiol*. doi:10.1007/s00127-023-02489-x

Ferguson, E., Dawe-Lane, E., Khan, Z., Reynolds, C., Davison, K., Edge, D., & Brailsford, S.R. (2022). [Trust and distrust: Identifying recruitment targets for ethnic minority blood donors](https://onlinelibrary.wiley.com/doi/epdf/10.1111/tme.12867) *Transfusion Medicine,* 32, 276–287 (doi. 10.1111/tme.12867)

Schoemann, A. M., Boulton, A. J., & Short, S. D. (2017). Determining power and sample size for simple and complex mediation models. Social Psychological and Personality Science, 8(4), 379–386. [https://doi.org/10.1177/1948550617715068](https://psycnet.apa.org/doi/10.1177/1948550617715068)

Shariff-Marco S, Breen N, Landrine H, et al. MEASURING EVERYDAY RACIAL/ETHNIC DISCRIMINATION IN HEALTH SURVEYS: How Best to Ask the Questions, in One or Two Stages, Across Multiple Racial/Ethnic Groups?. *Du Bois Rev*. 2011;8(1):159-177. doi:10.1017/S1742058X11000129

# Supplementary File S6: Percentages Agree, Neutral, and Disgaree by Ethnicity and Donor Status

We explore if avoidance and negative feedback were more likely to be observed within any community. To do this, we grouped responses for the HRP-SSA and Travel questions into three combined categories: (i) ‘strongly disagree/disagree’, (ii) ‘neither’, and (iii) ‘strongly agree/agree’. Table S3 shows the percentage responses in these three ethnic categories for the whole sample, current donors, and non-donors. For the HRP-SSA and Travel questions, White people were significantly more likely to ‘strongly disagree/disagree’ with the statement “Would put me off wanting to donate blood”, with Black people significantly more likely to ‘strongly agree/agree’, with this, as high as 34% of Black non-donors. In terms of negative feedback, White people were significantly more likely to ‘strongly disagree/disagree’ with the statement “Makes me want to tell others not to donate” with Black people significantly more likely to ‘strongly agree/agree’ than expected by chance, with this as high as 17.4% for Black non-donors. These findings support hypothesis H1.

## ***Table S3***. *Percentage Agreement and Disagree with Avoidance and _N_WOM.*

|  |  | “had sex with anyone who may ever have had sex in parts of the world where AIDS/HIV is very common (including most countries in Africa)?” | | | | | | | | | |
| --- | --- | --- | --- | --- | --- | --- | --- | --- | --- | --- | --- |
|  |  | Would put me off wanting to donate blood [**Avoidance**] | | | | | Makes me want to tell others not to donate [**_N_WOM**] | | | | |
|  |  | Strongly Disagree/Disagree | Neither | Strongly Agree/ Agee | *n* | χ2 | Strongly Disagree/Disagree | Neither | Strongly Agree/ Agee | *n* | χ2 |
| Whole Sample | Asian | 84.1% | 8.2% | 7.7% | 182 | 33.38*** | 90.1% | 6.0% | 8.3% | 182 | 43.39*** |
|  | Black | 70.9% | 8.5% | 20.6% | 141 |  | 75.9% | 14.2% | 9.9% | 141 |  |
|  | Mixed | 83.5% | 6.6% | 9.9% | 182 |  | 90.6% | 6.1% | 3.3% | 181 |  |
|  | White | 88.5% | 5.9% | 5.5% | 454 |  | 93.6% | 5.7% | 0.7% | 453 |  |
| Current Donors | Asian | 87.5% | 6.3% | 6.3% | 144 | 9.21 | 92.4% | 4.2% | 3.5% | 144 | 16.17* |
|  | Black | 83.0% | 4.5% | 12.5% | 88 |  | 83.0% | 11.4% | 5.7% | 88 |  |
|  | Mixed | 84.5% | 6.5% | 9.0% | 155 |  | 91.6% | 5.8% | 2.6% | 155 |  |
|  | White | 90.9% | 3.7% | 5.4% | 353 |  | 94.6% | 4.5% | 0.8% | 353 |  |
| Non-Donors | Asian | 71.1% | 15.8% | 13.2% | 38 | 23.58*** | 81.6% | 13.2% | 5.3% | 38 | 22.43*** |
|  | Black | 50.9% | 15.1% | 34.0% | 53 |  | 64.2% | 18.9% | 17.0% | 53 |  |
|  | Mixed | 77.8% | 7.4% | 14.8% | 27 |  | 84.6% | 7.7% | 7.7% | 26 |  |
|  | White | 80.2% | 13.9% | 5.9% | 101 |  | 90.0% | 10% | 0% | 100 |  |
|  |  | Travelled outside the UK and returned from an area where there is malaria, including many parts of Africa, Asia and South America | | | | | | | | | |
| Whole Sample | Asian | 90.1% | 6.1% | 3.9% | 181 | 47.84*** | 91.7% | 6.1% | 2.2% | 181 | 56.16*** |
|  | Black | 77.3% | 9.9% | 12.8% | 121 |  | 78.7% | 12.1% | 9.2% | 141 |  |
|  | Mixed | 92.3% | 5.0% | 2.7% | 181 |  | 95.1% | 3.8% | 1.1% | 182 |  |
|  | White | 94.9% | 3.6% | 1.6% | 450 |  | 94.7% | 5.1% | 0.2% | 449 |  |
| Current Donors | Asian | 92.3% | 4.3% | 3.5% | 143 | 24.08*** | 92.3% | 7.0% | 0.7% | 143 | 33.39*** |
|  | Black | 83.0% | 6.8% | 10.2% | 88 |  | 83.0% | 9.1% | 8.0% | 88 |  |
|  | Mixed | 93.5% | 4.5% | 2.0% | 154 |  | 94.8% | 3.9% | 1.3% | 155 |  |
|  |  | 90.6% | 2.6% | 1.4% | 351 |  | 94.9% | 4.9% | 0.3% | 350 |  |
| Non-Donors | Asian | 81.6% | 13.2% | 5.3% | 38 | 16.33* | 89.5% | 2.6% | 7.9% | 38 | 23.26*** |
|  | Black | 67.9% | 15.1% | 17.0% | 53 |  | 71.7% | 17.0% | 11.3% | 53 |  |
|  | Mixed | 85.2% | 7.4% | 7.4% | 27 |  | 96.3% | 3.7% | 0% | 27 |  |
|  | White | 90.9% | 7.1% | 2.0% | 99 |  | 93.9% | 6.1% | 0% | 99 |  |

* p < .05, ** p < .01. *** p < .001

# Supplementary File S7: Mediation Coefficients

Below in Table S4 are the indirect effects of ethnicity on outcomes via perceived racial discrimination in the NHS and [perceived social isolation. For people from all ethnic minority communities, there is a significant indirect effect on both avoidance and negative feedback via perceptions of higher racial discrimination but no indirect effects of social inclusion.

## ***Table S4***. Indirect Effects of Ethnicity on Avoidance and _N_WOM via Perceived Racial Discrimination (M_1_] and Perceived Social Inclusion [M_2_]

|  |  | Perceived Racial Discrimination [M_1_] | | | | | | | | | | | | |
| --- | --- | --- | --- | --- | --- | --- | --- | --- | --- | --- | --- | --- | --- | --- |
|  |  | Effect | 95% CI |  | Effect | 95% CI |  |  |  |  |  |  |  |  |
|  | [Y] Ethnicity | Indirect Effect (Sex) | Lower | Upper | Indirect Effect (Travel) | Lower | Upper |  | Indirect Effect (Sex) | Lower | Upper | Indirect Effect (Travel) | Lower | Upper |
| Avoidance [X] |  |  |  |  |  |  |  | _N_WOM  [X] |  |  |  |  |  |  |
|  | Mixed | **0.0325** | **0.0040** | **0.0703** | **0.0351** | **0.0070** | **0.0692** |  | **0.0169** | **0.0011** | **0.0402** | **0.0388** | **0.0066** | **0.0818** |
|  | Asian | **0.0434** | **0.0133** | **0.0823** | **0.0456** | **0.0158** | **0.0827** |  | **0.0229** | **0.0034** | **0.0484** | **0.0519** | **0.0177** | **0.0965** |
|  | Black | **0.1139** | **0.0529** | **0.1838** | **0.1173** | **0.0643** | **0.0177** |  | **0.0601** | **0.0127** | **0.1122** | **0.1005** | **0.0501** | **0.1633** |
|  |  | Perceived Social Inclusion [M_2_] | | | | | | | | | | | | |
| Avoidance [X] |  |  |  |  |  |  |  | _N_WOM  [X] |  |  |  |  |  |  |
|  | Mixed | 0.0037 | -0.0062 | 0.0184 | 0.0040 | -0.0063 | 0.0184 |  | 0.0043 | -0.0057 | 0.0188 | 0.0028 | -0.0044 | 0.0141 |
|  | Asian | -0.0049 | -0.0203 | 0.0050 | -0.0064 | -0.0212 | 0.0040 |  | -0.0059 | -0.0203 | 0.0040 | -0.0043 | -0.0175 | 0.0035 |
|  | Black | 0.0111 | -0.0054 | 0.0353 | 0.0131 | -0.0003 | 0.0335 |  | 0.0137 | -0.0004 | 0.0343 | 0.0089 | -0.0041 | 0.0268 |

***Note***. Significant effects are in bold, Comparison = White.

# Supplementary File S8: Moderation of Ethnicity Effects on Avoidance and Negative Feedback by Donor Status

## ***Table S5*** *Moderation of Ethnicity on Avoidance and Negative Feedback by Donor Status for Having Sex with Someone who may have had sex with someone in an HIV-Endemic area*

|  |  | Robust |  |  | 95% CI | |
| --- | --- | --- | --- | --- | --- | --- |
|  | Coefficient | std. err. | z | P = | Lower. | Upper |
| **Outcome**: **Avoidance** (Would put me off wanting to donate blood) |  |  |  |  |  |  |
| *Ethnicity* |  |  |  |  |  |  |
| Mixed | .3138462 | .2289375 | 1.37 | 0.170 | -.134863 | .7625554 |
| Asian | .0547368 | .2012669 | 0.27 | 0.786 | -.339739 | .4492127 |
| Black | **.8015094** | **.2145577** | **3.74** | **0.000** | **.380984** | **1.222035** |
| Current Donor (yes) | **-.2989235** | **.1053945** | **-2.84** | **0.005** | **-.505493** | **-.0923541** |
|  |  |  |  |  |  |  |
| Ethnicity by Donor Status |  |  |  |  |  |  |
| Mixed*yes | -.0936323 | .2481453 | -0.38 | 0.706 | -.5799881 | .3927234 |
| Asian*yes | .0152978 | .2199402 | 0.07 | 0.945 | -.415777 | .4463726 |
| Black*yes | -.4448586 | .2487917 | -1.79 | 0.074 | -.9324814 | .0427641 |
| Constant | 1.84 | .0945727 | 19.46 | 0.000 | 1.654641 | 2.025359 |
|  |  |  |  |  |  |  |
| **Outcome**: **_N_WOM**  (Makes me want to tell others not to donate) |  |  |  |  |  |  |
| ETHNICITY |  |  |  |  |  |  |
| Mixed | **.5969231** | **.2067123** | **2.89** | **0.004** | **.1917745** | **1.002072** |
| Asian | .1515789 | .1607943 | 0.94 | 0.346 | -.163572 | .4667299 |
| Black | **.8030189** | **.1803679** | **4.45** | **0.000** | **.4495043** | **1.156533** |
| Current Donor (yes) | **-.1145609** | **.0751697** | **-1.52** | **0.128** | **-.2618907** | **.0327689** |
| Ethnicity by Donor Status |  |  |  |  |  |  |
| Mixed*yes | **-.4849428** | **.2179265** | **-2.23** | **0.026** | **-.9120709** | **-.0578147** |
| Asian*yes | -.0239625 | .1770571 | -0.14 | 0.892 | -.370988 | .3230631 |
| Black*yes | **-.5207307** | **.2085938** | **-2.50** | **0.013** | **-.929567** | **-.1118944** |
| Constant | 1.48 | .0670522 | 22.07 | 0.000 | 1.34858 | 1.61142 |

***Figure S1***. *Moderation of _N_WOM by Donor Status and Ethnicity*


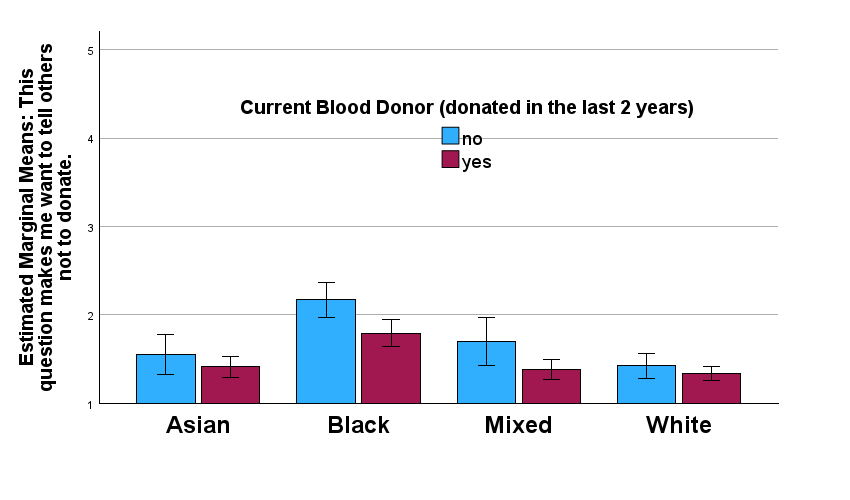


Figure S1 above shows that the significant interactions between ethnicity and donor status in Table S2 on negative feedback are attributable to Black and Mixed donors being less likely to tell others not to donate than Black and Mixed non-donors.

## ***Table S6***. *Moderation of Ethnicity on Avoidance and _N_WOM by Donor Status for Travel to a Malarious Area*

|  |  | Robust |  |  | 95% CI | |
| --- | --- | --- | --- | --- | --- | --- |
|  | Coefficient | SE | z | P= | Lower | Upper |
| **Outcome**: **Avoidance** (Would put me off wanting to donate blood) |  |  |  |  |  |  |
| *Ethnicity* |  |  |  |  |  |  |
| Mixed | **.3367003** | **.1699941** | **1.98** | **0.048** | **.003518** | **.6698827** |
| Asian | .0316321 | .1636689 | 0.19 | 0.847 | -.2891531 | .3524174 |
| Black | **.6378883** | **.1750902** | **3.64** | **0.000** | **.2947179** | **.9810587** |
| Current Donor (yes) | **-.2776912** | **.0819682** | **-3.39** | **0.001** | **-.4383458** | **-.1170366** |
| Ethnicity by Donor Status |  |  |  |  |  |  |
| Mixed*yes | -.1917653 | .1822443 | -1.05 | 0.293 | -.5489575 | .165427 |
| Asian*yes | .0743419 | .1787686 | 0.42 | 0.678 | -.2760381 | .424722 |
| Black*yes | -.1796416 | .2069175 | -0.87 | 0.385 | -.5851923 | .2259092 |
| Constant | 1.626263 | .0750279 | 21.68 | 0.000 | 1.479211 | 1.773315 |
|  |  |  |  |  |  |  |
| **Outcome**: **_N_WOM** (Makes me want to tell others not to donate) |  |  |  |  |  |  |
| *Ethnicity* |  |  |  |  |  |  |
| Mixed | **.2794613** | **.1189854** | **2.35** | **0.019** | **.0462541** | **.5126684** |
| Asian | .1283892 | .1550745 | 0.83 | 0.408 | -.1755513 | .4323297 |
| Black | **.7455689** | **.1664676** | **4.48** | **0.000** | **.4192983** | **1.071839** |
| Current Donor (yes) | -.0870996 | .0685978 | -1.27 | 0.204 | -.2215489 | .0473497 |
| Ethnicity by Donor Status |  |  |  |  |  |  |
| Mixed*yes | -.2269937 | .1350324 | -1.68 | 0.093 | -.4916524 | .037665 |
| Asian*yes | -.0529446 | .1674169 | -0.32 | 0.752 | -.3810756 | .2751864 |
| Black*yes | -.2872572 | .2009689 | -1.43 | 0.153 | -.6811489 | .1066345 |
| Constant | 1.424242 | .0607589 | 23.44 | 0.000 | 1.305157 | 1.543328 |

# Supplementary File S9: Moderation by Family Member Being a Donor on Travel with Respect to Donor Status

## ***Non-Current Donors Travel***

## ***Table S7*** *Moderation of Ethnicity on Avoidance and _N_WOM by Donor Status for Travel to a Malarious Area (Non-Donors)*

|  |  | Robust |  |  | 95% CI | |
| --- | --- | --- | --- | --- | --- | --- |
|  | Coefficient | std. err. | z | P= | Lower. | Upper |
| **Outcome**: **Avoidance** (Would put me off wanting to donate blood) |  |  |  |  |  |  |
| *Ethnicity* |  |  |  |  |  |  |
| Mixed | **.6100168** | **.2014223** | **3.03** | **0.002** | .2152364 | 1.004797 |
| Asian | .2236848 | .2025399 | 1.10 | 0.269 | -.1732861 | .6206557 |
| Black | **.8726313** | **.2426997** | **3.60** | **0.000** | .3969486 | 1.348314 |
| Know Family Member Who Donates Blood (Yes) | **.4417599** | **.1487621** | **2.97** | **0.003** | .1501915 | .7333283 |
| Ethnicity By Knowing Family Member Who Donates Blood |  |  |  |  |  |  |
| Mixed*yes | -.4912947 | .3794568 | -1.29 | 0.195 | -1.235016 | .2524269 |
| Asian*yes | -.3316123 | .3333027 | -0.99 | 0.320 | -.9848736 | .3216491 |
| Black*yes | -.3252382 | .3570433 | -0.91 | 0.362 | -1.02503 | .3745538 |
| Gender (male) | .10943 | .1600512 | 0.68 | 0.494 | -.2042647 | .4231246 |
| Age | .0018669 | .0047316 | 0.39 | 0.693 | -.0074069 | .0111408 |
| Constant | 1.26991 | .2788079 | 4.55 | 0.000 | .7234568 | 1.816364 |
|  |  |  |  |  |  |  |
| **Outcome**: **_N_WOM**  (Makes me want to tell others not to donate) |  |  |  |  |  |  |
| *Ethnicity* |  |  |  |  |  |  |
| Mixed | **.4794325** | **.1610034** | **2.98** | **0.003** | **.1638716** | **.7949934** |
| Asian | .3555462 | .2271274 | 1.57 | 0.117 | -.0896154 | .8007078 |
| Black | **.8713705** | **.2193596** | **3.97** | **0.000** | **.4414336** | **1.301307** |
| Know Family Member Who Donates Blood (Yes) | **.284668** | **.1317907** | **2.16** | **0.031** | **.026363** | **.5429731** |
| Ethnicity By Know Family Member Who Donates Blood |  |  |  |  |  |  |
| Mixed*yes |  |  |  |  |  |  |
| Asian*yes | -.1407599 | .2480579 | -0.57 | 0.570 | -.6269445 | .3454247 |
| Black*yes | -.2729462 | .3006691 | -0.91 | 0.364 | -.8622468 | .3163544 |
| Gender (male) | .150743 | .3409414 | 0.44 | 0.658 | -.51749 | .8189759 |
| Age | .1955878 | .1613534 | 1.21 | 0.225 | -.120659 | .5118346 |
| Know Family Member Who Donates Blood (Yes) | .0099869 | .0044078 | 2.27 | 0.023 | .0013477 | .0186261 |
| Constant | .7304045 | .2584272 | 2.83 | 0.005 | .2238965 | 1.236912 |

## ***Current-Donors Travel***

## ***Table S8***. *Moderation of Ethnicity on Avoidance and _N_WOM* *by Donor Status for Travel to a Malarious Area (Current Donors)*

|  |  | Robust |  |  | 95% CI | |
| --- | --- | --- | --- | --- | --- | --- |
|  | Coefficient | std. err. | z | P= | Lower | Upper |
| **Outcome**: **Avoidance** (Would put me off wanting to donate blood) |  |  |  |  |  |  |
| *Ethnicity* |  |  |  |  |  |  |
| Mixed | .1907149 | .1196801 | 1.59 | 0.111 | -.0438537 | .4252835 |
| Asian | .1010742 | .1328254 | 0.76 | 0.447 | -.1592587 | .3614072 |
| Black | **.4302849** | **.1657496** | **2.60** | **0.009** | **.1054217** | **.7551481** |
| Know Family Member Who Donates Blood (Yes) | .0051968 | .0771589 | 0.07 | 0.946 | -.1460318 | .1564254 |
| Ethnicity By Knowing Family Member Who Donates Blood |  |  |  |  |  |  |
| Mixed*yes | -.1420525 | .1411687 | -1.01 | 0.314 | -.4187381 | .134633 |
| Asian*yes | -.1024905 | .150036 | -0.68 | 0.495 | -.3965556 | .1915746 |
| Black*yes | .0865108 | .2312224 | 0.37 | 0.708 | -.3666769 | .5396984 |
| Gender (male) | .0577022 | .0576134 | 1.00 | 0.317 | -.0552181 | .1706224 |
| Age | -.0022217 | .0020549 | -1.08 | 0.280 | -.0062492 | .0018058 |
| Constant | 1.434347 | .1266094 | 11.33 | 0.000 | 1.186197 | 1.682497 |
|  |  |  |  |  |  |  |
| **Outcome**: **_N_WOM**  (Makes me want to tell others not to donate) |  |  |  |  |  |  |
| *Ethnicity* |  |  |  |  |  |  |
| Mixed | .1980765 | .118136 | 1.68 | 0.094 | -.0334659 | .4296188 |
| Asian | .045352 | .0969842 | 0.47 | 0.640 | -.1447337 | .2354376 |
| Black | **.5306032** | **.1677689** | **3.16** | **0.002** | **.2017823** | **.8594241** |
| Know Family Member Who Donates Blood (Yes) | .0352778 | .0698244 | 0.51 | 0.613 | -.1015756 | .1721312 |
| Ethnicity By Know Family Member Who Donates Blood |  |  |  |  |  |  |
| Mixed*yes | -.2342984 | .1340436 | -1.75 | 0.080 | -.497019 | .0284222 |
| Asian*yes | .103327 | .13208 | 0.78 | 0.434 | -.1555451 | .362199 |
| Black*yes | -.0349662 | .2334274 | -0.15 | 0.881 | -.4924754 | .4225431 |
| Gender (male) | -.0086555 | .0548851 | -0.16 | 0.875 | -.1162283 | .0989173 |
| Age | .0022612 | .0020283 | 1.11 | 0.265 | -.0017142 | .0062367 |
| Constant | 1.202583 | .116439 | 10.33 | 0.000 | .9743662 | 1.430799 |

# Supplementary File S10: Moderation by Family Member Being a Donor on Sex with Respect to Donor Status

## ***Non-Current Donors Sex***

## ***Table S9***. *Moderation of Ethnicity on Avoidance and _N_WOM by Donor Status for Sex (Non-Donors)*

|  |  | Robust |  |  | 95% CI | |
| --- | --- | --- | --- | --- | --- | --- |
|  | Coefficient | std. err. | z | P= | Lower | Upper |
| **Outcome**: **Avoidance** (Would put me off wanting to donate blood) |  |  |  |  |  |  |
| *Ethnicity* |  |  |  |  |  |  |
| Mixed | .4191653 | .2982662 | 1.41 | 0.160 | -.1654256 | 1.003756 |
| Asian | .0428512 | .2733109 | 0.16 | 0.875 | -.4928282 | .5785307 |
| Black | **1.017046** | **.296009** | **3.44** | **0.001** | **.4368787** | **1.597213** |
| Know Family Member Who Donates Blood (Yes) | **.3727566** | **.1991989** | **1.87** | **0.061** | **-.017666** | **.7631793** |
| Ethnicity By Know Family Member Who Donates Blood |  |  |  |  |  |  |
| Mixed*yes | -.1350103 | .5095374 | -0.26 | 0.791 | -1.133685 | .8636646 |
| Asian*yes | -.0197939 | .3959775 | -0.05 | 0.960 | -.7958956 | .7563077 |
| Black*yes | -.3776867 | .4338847 | -0.87 | 0.384 | -1.228085 | .4727116 |
| Gender (male) | .2855198 | .1943526 | 1.47 | 0.142 | -.0954043 | .6664439 |
| Age | .002236 | .0060191 | 0.37 | 0.710 | -.0095613 | .0140332 |
| Constant | 1.49525 | .3616204 | 4.13 | 0.000 | .7864875 | 2.204013 |
|  |  |  |  |  |  |  |
| **Outcome**: **_N_WOM**  (Makes me want to tell others not to donate) |  |  |  |  |  |  |
| *Ethnicity* |  |  |  |  |  |  |
| Mixed | .6763658 | .2650321 | 2.55 | 0.011 | .1569125 | 1.195819 |
| Asian | .2528881 | .2162526 | 1.17 | 0.242 | -.1709591 | .6767353 |
| Black | **.8613479** | **.2347882** | **3.67** | **0.000** | **.4011716** | **1.321524** |
| Know Family Member Who Donates Blood (Yes) | .2275507 | .1439576 | 1.58 | 0.114 | -.0546009 | .5097024 |
| Ethnicity By Knowing Family Member Who Donates Blood |  |  |  |  |  |  |
| Mixed*yes | .0170283 | .4667342 | 0.04 | 0.971 | -.8977538 | .9318105 |
| Asian*yes | -.1410655 | .3154998 | -0.45 | 0.655 | -.7594337 | .4773027 |
| Black*yes | .1443962 | .3786267 | 0.38 | 0.703 | -.5976986 | .8864909 |
| Gender (male) | .2494283 | .1796435 | 1.39 | 0.165 | -.1026664 | .601523 |
| Age | .0071951 | .0046558 | 1.55 | 0.122 | -.0019302 | .0163203 |
| Constant | .9647206 | .2745393 | 3.51 | 0.000 | .4266334 | 1.502808 |

## ***Current-Donors Sex***

## ***Table S10***. *Moderation of Ethnicity on Avoidance and _N_WOM by Donor Status for Sex (Current Donors)*

|  |  | Robust |  |  | 95% CI | |
| --- | --- | --- | --- | --- | --- | --- |
|  | Coefficient | std. err. | z | P= | Lower | Upper |
| **Outcome**: **Avoidance** (Would put me off wanting to donate blood) |  |  |  |  |  |  |
| *Ethnicity* |  |  |  |  |  |  |
| Mixed | **.3096544** | **.1552694** | **1.99** | **0.046** | **.005332** | **.6139768** |
| Asian | -.0189905 | .130235 | -0.15 | 0.884 | -.2742464 | .2362653 |
| Black | .2005023 | .1426256 | 1.41 | 0.160 | -.0790387 | .4800432 |
| Know Family Member Who Donates Blood (Yes) | .1027901 | .0898426 | 1.14 | 0.253 | -.0732982 | .2788785 |
| Ethnicity By Knowing Family Member Who Donates Blood |  |  |  |  |  |  |
| Mixed*yes | -.2715115 | .1915291 | -1.42 | 0.156 | -.6469016 | .1038786 |
| Asian*yes | .0606005 | .174289 | 0.35 | 0.728 | -.2809997 | .4022008 |
| Black*yes | .4391685 | .2736989 | 1.60 | 0.109 | -.0972716 | .9756085 |
| Gender (male) |  |  |  |  |  |  |
| Age | .0176591 | .0701413 | 0.25 | 0.801 | -.1198153 | .1551335 |
| Know Family Member Who Donates Blood (Yes) | -.0047191 | .002713 | -1.74 | 0.082 | -.0100366 | .0005983 |
| Constant | 1.680846 | .1620482 | 10.37 | 0.000 | 1.363237 | 1.998455 |
|  |  |  |  |  |  |  |
| **Outcome**: **_N_WOM** (Makes me want to tell others not to donate) |  |  |  |  |  |  |
| *Ethnicity* |  |  |  |  |  |  |
| Mixed | .170342 | .1043079 | 1.63 | 0.102 | -.0340976 | .3747816 |
| Asian | .1037271 | .1041848 | 1.00 | 0.319 | -.1004714 | .3079255 |
| Black | **.2801992** | **.1350813** | **2.07** | **0.038** | **.0154447** | **.5449536** |
| Know Family Member Who Donates Blood (Yes) | .0962484 | .0712076 | 1.35 | 0.176 | -.0433159 | .2358128 |
| Ethnicity By Knowing Family Member Who Donates Blood |  |  |  |  |  |  |
| Mixed*yes | -.1301272 | .140322 | -0.93 | 0.354 | -.4051533 | .144899 |
| Asian*yes | .0136894 | .146993 | 0.09 | 0.926 | -.2744117 | .3017905 |
| Black*yes | .150361 | .2250517 | 0.67 | 0.504 | -.2907323 | .5914543 |
| Gender (male) | .0193439 | .0552063 | 0.35 | 0.726 | -.0888584 | .1275462 |
| Age | -.0006853 | .002128 | -0.32 | 0.747 | -.0048562 | .0034856 |
| Constant | 1.319087 | .1224191 | 10.78 | 0.000 | 1.07915 | 1.559024 |

# Supplementary Files S11: Epidemiology Tables

## ***Table S11:*** ***Number of recently acquired infections (<12m) in blood donors, looking at area sexually acquired, UK 2015-2019***

| Number | Recent infection | Sexually acquired | Sex UK | Sex Thailand | Sex Europe | | Sex other | | Country nk |
| --- | --- | --- | --- | --- | --- | --- | --- | --- | --- |
| HBV | 17 | **11** | 6 | 3 | | 2 | | 0 | 0 |
| HCV | 1 | **0** |  |  | |  | |  |  |
| HIV | 12 | **11** | 8 | 1 | | 2 | | 0 | 0 |
| Syphilis | 135 | **123** | 101 | 1 | | 5 | | 2 | 14 |
